# Predicting Global Fund grant disbursements for procurement of artemisinin-based combination therapies

**DOI:** 10.1186/1475-2875-7-200

**Published:** 2008-10-02

**Authors:** Justin M Cohen, Inder Singh, Megan E O'Brien

**Affiliations:** 1 Clinton Foundation HIV/AIDS Initiative, Center for Strategic HIV Operations Research (CSHOR), 383 Dorchester Avenue, Suite 400, Boston, MA 02127, USA; 2 Clinton Foundation HIV/AIDS Initiative, Drug Access Team, 383 Dorchester Avenue, Suite 400, Boston, MA 02127, USA

## Abstract

**Background:**

An accurate forecast of global demand is essential to stabilize the market for artemisinin-based combination therapy (ACT) and to ensure access to high-quality, life-saving medications at the lowest sustainable prices by avoiding underproduction and excessive overproduction, each of which can have negative consequences for the availability of affordable drugs. A robust forecast requires an understanding of the resources available to support procurement of these relatively expensive antimalarials, in particular from the Global Fund, at present the single largest source of ACT funding.

**Methods:**

Predictive regression models estimating the timing and rate of disbursements from the Global Fund to recipient countries for each malaria grant were derived using a repeated split-sample procedure intended to avoid over-fitting. Predictions were compared against actual disbursements in a group of validation grants, and forecasts of ACT procurement extrapolated from disbursement predictions were evaluated against actual procurement in two sub-Saharan countries.

**Results:**

Quarterly forecasts were correlated highly with actual smoothed disbursement rates (r = 0.987, p < 0.0001). Additionally, predicted ACT procurement, extrapolated from forecasted disbursements, was correlated strongly with actual ACT procurement supported by two grants from the Global Fund's first (r = 0.945, p < 0.0001) and fourth (r = 0.938, p < 0.0001) funding rounds.

**Conclusion:**

This analysis derived predictive regression models that successfully forecasted disbursement patterning for individual Global Fund malaria grants. These results indicate the utility of this approach for demand forecasting of ACT and, potentially, for other commodities procured using funding from the Global Fund. Further validation using data from other countries in different regions and environments will be necessary to confirm its generalizability.

## Background

Since the 1970s, the rise of global resistance to the cheap, ubiquitous antimalarial chloroquine (CQ) has made it imperative to find new, effective drugs to fight *Plasmodium falciparum*, the parasite species responsible for a majority of malaria-related mortality worldwide. Modern artemisinin drugs were first developed by the Chinese for the Viet Cong during the Vietnam-American War, on the basis of ancient Chinese fever remedies involving *Artemisia annua*, the sweet wormwood plant. Artemisinin-based combination therapy (ACT), which utilizes artemisinin along with an additional partner drug, such as amodiaquine, lumefantrine or mefloquine, is now widely considered the most effective treatment for uncomplicated malaria [1].

Following a 2003 critique in the Lancet [2], the WHO revised its malaria treatment guidelines to recommend ACT over all other therapies [1], and the Global Fund to Fight AIDS, Tuberculosis and Malaria ("the Global Fund") began to support acceleration of ACT scale-up actively [3]. The ensuing sudden upsurge in demand for artemisinin caused large fluctuations in the price and supply of artemisinin. An initially inadequate supply of this key ingredient resulted in a sharp price increase up to $1,200 per kg in 2004–2005, but prices then plunged to as low as $170 per kg in 2007 following subsequent overproduction [4]. Accurate forecasts are needed to stabilize the market by ensuring that production reflects demand [5], but such efforts are complicated by the uncertainty and opacity of global procurement operations. The assessment of the forecasting landscape by a 2004 Institute of Medicine report was bleak: "*There is widespread agreement that accurate forecasts of demand are essential to the success of procurement operations. Agreement is equally widespread that accurately forecasting the demand for antimalarials is nearly impossible*"[6].

The environment surrounding forecasting today remains dynamic, with evolution of national treatment guidelines [7] and continued deployment of new ACT products [8]. The potential implementation of a global subsidy under the Roll Back Malaria (RBM) Affordable Medicines Facility for Malaria has further complicated efforts, since such a subsidy has the potential to cause substantial increases in demand for ACT, through a dramatic reduction of the price of these products [6]. For forecasting efforts to maintain credibility under such dynamic circumstances, they must be adaptable and robust, with explicit and transparent specification of underlying assumptions, source data, and methodology.

Accurate forecasting of demand is critical for ACT, because the key ingredient, artemisinin, is a natural product derived from a plant, creating supply chain challenges [9]. Production of an ACT requires 12 to 24 months from the planting of *Artemisia annua *to ACT production [4]. This timeline compares to three to five months for either fully synthetic drugs (e.g., antiretrovirals or tuberculosis drugs) or semi-synthetic drugs requiring fermentation processes (e.g., some antibiotics). Given the lengthy lead time required, accurate forecasting of procurement is critical to ensure access to high-quality, life-saving ACTs at the lowest sustainable prices [6] by avoiding the twin dangers of underproduction – which would lead to shortages and higher prices – and excessive overproduction – which would lead to expiry of these drugs, which have a shelf-life of about two years [8], and losses for suppliers that might drive some out of the market and reduce incentives for others to enter it.

ACT is significantly more expensive than conventional antimalarial medicines [8], necessitating support from donor organizations for purchase by resource-limited governments. Because of the importance of this funding, accurate forecasting of ACT procurement requires understanding the dynamics of the donor funding mechanisms. The Global Fund is presently the single largest funding source for ACT procurement worldwide, funding an estimated 264 million treatments through its fifth funding round [10]. Analysis of global ACT financing indicates that the Global Fund is responsible for funding approximately 70–78% of all public sector ACT procurement [11]. Funding of ACT through non-Global Fund mechanisms by other actors, including the United States President's Malaria Initiative, UNITAID, and the World Bank, while significant, comprises a much smaller share of global volumes. Given that the Global Fund drivers a majority of the world's ACT volumes, careful understanding and robust prediction of its funding process will comprise an essential component of an accurate global demand forecast.

During each round, countries apply to the Global Fund for funding by submitting a proposal. Funding for approved proposals is transmitted to recipient countries through a series of disbursements. The magnitude and timing of each disbursement of funding is contingent upon the country's performance in meeting key milestones (e.g., distribution of procured ACT) as stated in the approved grant agreement, as well as the country's compliance with the Global Fund's policies. Disbursement of awarded funding may slow or be halted if a country's progress is deemed insufficient. Thus, it is hypothesized that the rate at which a grant is disbursed can be considered an indicator of grant progress. For example, if a substantial portion of a particular grant is intended for ACT procurement, a large disbursement rate may be considered indicative of successful procurement (although performance of all grant components is taken into consideration when determining grant disbursements), and the magnitude and timing of that procurement can be estimated as a function of the supporting grant's disbursement rate.

Past research has investigated factors associated with grant performance. Lu *et al *[12] used random effects analyses to look for associations between characteristics of grants and recipients and quarterly disbursement of funds. They found that large grants had lower disbursement rates and grants with private principal recipients were disbursed more rapidly. Additionally, higher political stability indices, lower gross domestic product (GDP), and lower proportion of GDP spent on health all were associated with faster rates of disbursement. Radelet and Siddiqi [13] identified characteristics of the grant and recipient that were associated with evaluation scores (higher scores will be correlated with greater disbursement rates). The investigation found that poorly rated grants were more likely to have governmental principal recipients, be focused on malaria, have weak initial proposals, or be evaluated by particular firms, while having more doctors, higher measles immunization rate, few other donors, and high disease prevalence were associated with higher scores.

The analysis presented here describes the derivation of predictive regression models to forecast the rate at which malaria grant funding is disbursed from the Global Fund to recipient countries. The utility of these predictions for estimation of future ACT procurement then is demonstrated using importation records from two sub-Saharan African countries.

## Methods

**Figure 1:**
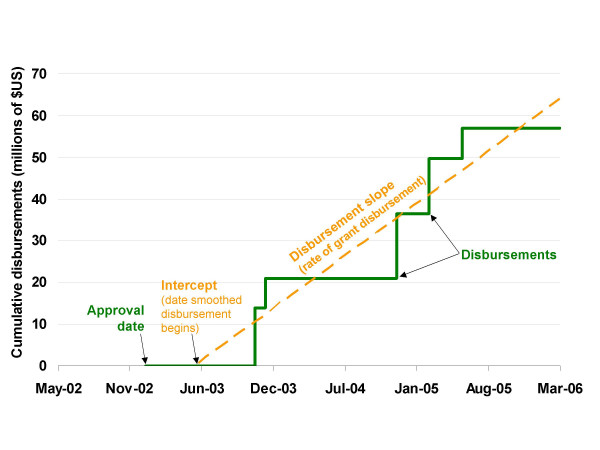
**Fitting disbursement slopes and intercepts**. Depiction of the ordinary least-squares regression used to calculate the disbursement slope and intercept for a hypothetical Global Fund grant.

Data on Global Fund grant awards [14] and disbursements [15] were downloaded from the Global Fund website for analysis. Historical disbursement of each Global Fund malaria grant awarded in funding Rounds 1–6 was represented by a simple linear regression line, fit using the date as the predictor variable and the daily cumulative disbursement amount (beginning the day the grant was approved and ending on either the date of grant completion or 1 January, 2008, whichever came first) as the outcome. From this line, two key parameters were defined (Figure 1):

(1) The rate at which funding was disbursed from the Global Fund to the recipient country (the disbursement slope)

(2) The date on which disbursement began (the disbursement line intercept)

### Model fitting

**Figure 2:**
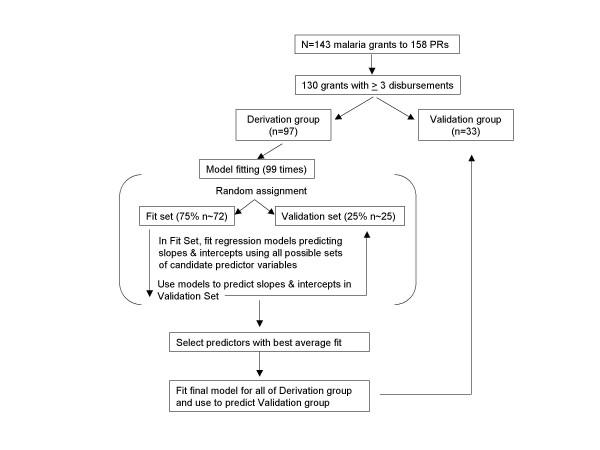
Diagram of split-sample model fitting methodology.

Predictive regression models may include variables that are not statistically significant and exclude others that are, since statistical association does not necessarily indicate useful predictive value [16]. To prevent over-fitting of models to the training dataset and to ensure that the variables included in models were not merely statistically significant but also demonstrated predictive ability [17,18], models were constructed using a repeated split-sample procedure (Figure 2). Only those grants for which the Global Fund had made at least three prior funding disbursements to the recipient country (n = 130) were used to fit and test the predictive models, since initial disbursements might not be indicative of future performance. A randomly selected 75% (n = 97) of these Round 1–6 grants were used to fit and compare models constructed with candidate variables (the "derivation group"), while the remaining 25% of grants (n = 33) were set aside during model fitting to permit validation on data not used to fit the model (the "validation group").

Two models were constructed, one to predict the disbursement slope, and the other to predict the intercept of the disbursement line. Variable selection for each model occurred through a series of 99 model fitting and testing repetitions (Figure 2). In each iteration, the 97 grants in the derivation group were subdivided into further random sets: 75% were assigned to a fitting set and 25% to a testing set. The fitting set was used to construct models involving different combinations of candidate variables; candidate predictor variables are listed in Table 1. These models then were compared according to their ability to predict the slope or intercept of the fit disbursement lines in the testing set. The best predicting set of variables was judged according to which produced the lowest root-mean-square error (RMSE) for each repetition. The sets of variables that produced the lowest average RMSE over all 99 repetitions were selected to construct final models.

**Table 1:** Candidate explanatory variables entered into predictive regression models

**Grant characteristics**	**Country characteristics**
Funding round	Number of previous malaria grants
Grant amount per populace	Number of previous grants of any type
Phase 1 and 2 potential grant amount	Population
Phase 1 and 2 agreed grant amount	World region
Principal Recipient (PR) type	Control of Corruption Index [24]
Local Fund Agent (LFA)	Political Stability Index [24]
Planned grant length	Government Effectiveness Index [24]
	Physicians per populace [25]
	Measles immunization rate for < 1 yr olds [25]
	Observer Human Rights Index [26]
	Estimated malaria cases [27]
	Under-five mortality rate [28]
	GDP per capita [29]

After variable selection, final models predicting slope and intercept were fit using all 97 grants in the derivation group. These multivariate regression models then were used to predict the disbursement slopes and intercepts for the unused 33 grants in the validation dataset. Each grant's predicted slope and intercept were used to generate a line representing estimated funding disbursements, extending until either reaching the grant's maximum potential or expiration date, whichever came first. The expiration date was calculated by adding the planned length of the grant to the grant start date. Predicted quarterly disbursements for 2003–2007 were summed across all grants, and these predictions were compared against summations of the actual disbursement lines fit to grant disbursement history [15] as well as the summation of planned funding from grant proposals [19].

### Summation of planned funding for malaria grants

To examine how actual grant performance differed from planned disbursement, the amount of funding requested by recipient countries from the Global Fund for each year of the grant was extracted from the 146 approved malaria grant proposals listed on the Global Fund website [19]. Since total proposed funding may not match the actual amounts subsequently awarded in all cases, each year's requested funding was normalized against the actual awarded total [14]. The starting date for each grant was used to mark the beginning of that grant's first year, and the expected quarterly funding for each successive year (up to five) thereafter was extracted. Total expected disbursements for each quarter were then calculated by summing expected funding for each grant, and these totals were compared against the actual disbursement rates as well as those forecasted by the predictive models.

### Estimating ACT procurement using forecasted disbursement

Each approved malaria Global Fund proposal was read to determine the proportion of each year's request intended for procurement of drugs for uncomplicated malaria. The percentage of the grant intended for procurement of drugs for uncomplicated malaria was then calculated separately for Phase 1 (years 1–2) and Phase 2 (years 3–5) of the grant, if applicable. If an itemized drug budget was not available in a particular proposal (39 proposals), average values were substituted from either the applicant country's other grant proposals, when possible, or otherwise from all grants in that funding round.

Multiplying predicted disbursements by the percentage intended for procurement of drugs for uncomplicated malaria resulted in a prediction of the amount of funding available for ACT procurement for each grant. Because ACT orders are customarily delivered to countries in staggered shipments over the course of a year, forecasted demand for ACT was lagged one year behind predicted disbursements from the Global Fund to recipient countries. For example, hypothetical country × might receive a disbursement of $1 M on 1 Jan 2005; of this $1 M, the Global Fund grant calls for 80% of 2005 funds to be used for purchase of drugs, and therefore, the country immediately places an $800,000 ACT order to cover yearly drug requirements. ACT arrives in staggered shipments throughout the year, until all $800,000 worth have been delivered by the end of 2005. Once the amount of funding for drugs had been predicted for each grant, amounts were summed across all grants for a particular country to determine predicted funding available for ACT procurement in that country, and globally, to examine the amount of ACT procurement the Global Fund may fund each year.

To validate predictions of country-specific ACT demand generated using this approach, data were collected on ACT importation supported by specific Global Fund grants. Validation requires a complete set of importation records for all the ACT procured using Global Fund grants, including the date of all deliveries, total volumes, and their prices. Although partial records were obtained from several countries, comprehensive importation records were compiled for only two grants: one awarded during the first funding round to a Southern African country and one awarded in Round 4 to a country in East Africa. Data on all ACT orders placed and shipments received were obtained from sources including the National Medical Stores, the National Drug Authorities, and the Ministries of Health or Malaria Control Programmes. Cumulative dollars spent on ACT over time were summed from these records and compared to forecasted procurement.

## Results

**Figure 3:**
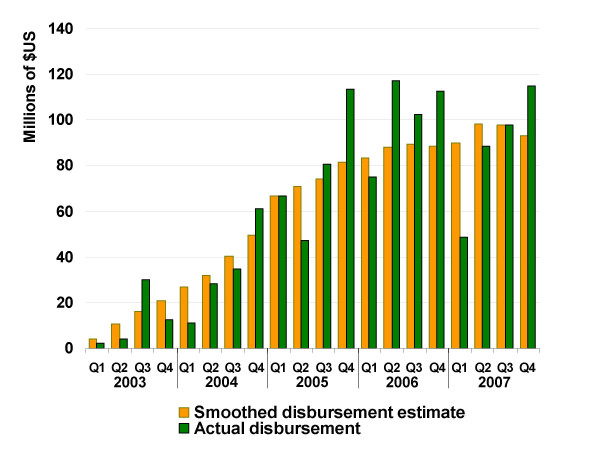
**Smoothed quarterly disbursements versus actual quarterly disbursements**. Comparison of smoothed quarterly approximations from regressing cumulative disbursements against time and actual quarterly disbursements on all Global Fund malaria grants from funding Round 1–6.

Through Round 7, 143 malaria grants had been awarded to 158 principal recipients (15 grants had two recipient organizations) in 75 countries or multi-country cooperatives. Fitting regression lines to each grant's cumulative disbursement resulted in smoothed quarterly estimates of total disbursements from the Global Fund to recipient countries (Figure 3). Smoothed quarterly estimates were correlated with actual disbursements, with r = 0.565 (p < 0.01). Three-quarters of Round 1–6 grants (no grants in Round 7 had begun disbursement at the time of analysis) with at least three disbursements (n = 97) were used in a repeated split-sample procedure to select best-predicting variables and fit final predictive regression models forecasting disbursement slopes and intercepts [see Additional Files – Additional File 1]. In these models, the disbursement slope was positively associated with the total agreed funding, the maximum potential size of phase one of the grant, being located in East Africa, the funding round in which the grant was awarded, and the World Bank's rating of that country's control of corruption. Slope was negatively associated with agreed funding for phase two of the grant.

In the final model predicting the intercept, or date on which funding was predicted to begin disbursement, earlier initiation of disbursement was associated with being located in the African or American WHO world regions, having a grant in a later funding round, being ranked by the World Bank with a better Government Effectiveness Index, and having a lower Observer Human Rights Index (fewer infractions). Disbursement began later for grants in the Southeast Asian WHO region, grants requested for longer periods of time (78% of Round 1–6 grants requested five years of funding, while the rest requested 2–4 years of support), and for countries with greater numbers of malaria grants.

**Figure 4:**
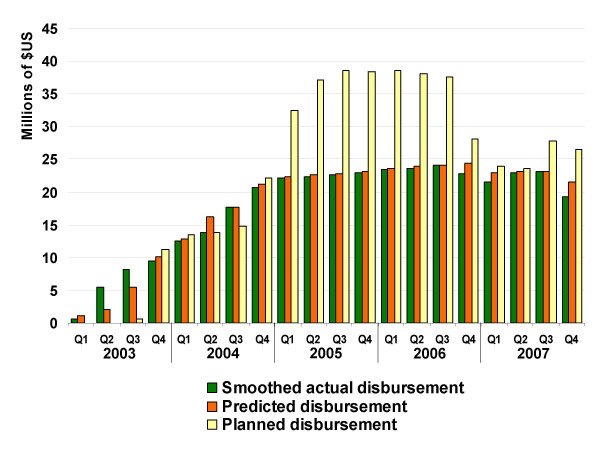
**Predicted quarterly disbursements**. Comparison of quarterly disbursements on 33 Global Fund malaria grants in the validation dataset as (1) calculated from the actual smoothed slopes, (2) estimated from predictive models, and (3) summed from disbursement planned in grant proposals.

Using these models, disbursement slopes and intercepts were predicted for the 33 grants in the validation set. These predictions were compared against the smoothed actual disbursement lines and the disbursements planned in the grant application (Figure 4). Predicted yearly global estimates averaged an error of +/- $3.05 M (6.7%) and were significantly correlated with the fit lines (r = 0.998, p < 0.0001). Yearly error ranged from $1.0 M (1.1%) in 2005 to $4.9 M (20.6%) in 2003. Quarterly percent error was higher than yearly error, with an average of +/- $875,503 (11.7%) per quarter, but quarterly estimates were still highly and significantly correlated with fit lines (r = 0.987, p < 0.0001). Quarterly estimates from the simple summation of planned funding were also significantly correlated with smoothed quarterly disbursements (r = 0.919, p < 0.0001), but were less accurate than the prediction model with an average error of +/- $7.0 M (43.5%) per quarter and +/- $28.1 M per year (40.9%).

### Predicted ACT procurement

The antimalarial procurement plans outlined in grant proposals were used to estimate the fraction of disbursements funding ACT procurement for each grant. Multiplying these fractions by the predicted yearly disbursement amounts for each funding round produced the round-specific global funding estimates depicted in Table 2.

**Table 2:** Predicted Global Fund disbursements for ACTs (in $US)

**Round**	**2003**	**2004**	**2005**	**2006**	**2007**	**2008**
**1**	174,062	3,292,628	3,394,641	3,411,586	3,104,795	3,025,509
**2**	-	5,511,019	11,737,573	11,574,975	9,066,550	7,012,194
**3**	-	38,876	2,614,055	3,007,743	2,959,491	2,384,943
**4**	-	-	4,231,787	64,863,377	65,816,864	66,073,528
**5**	-	-	-	1,866,298	16,332,993	16,657,853
**6**	-	-	-	-	1,657,285	11,358,460
**7**	-	-	-	-	-	2,666,991

**Total**	174,062	7,486,696	18,054,214	64,461,682	76,303,706	85,902,497

To examine whether the model could successfully predict actual procurement supported by individual Global Fund grants, ACT procurement predictions were validated against procurement records from two sub-Saharan countries, one supported by a Round 4 grant and the other by a Round 1 grant. Actual procurement records indicated that the Round 4 grant (Figure 3A) supported the importation of 20.7 M artemether-lumefantrine treatments, with a value of US $30.1 M, as of 27 June 2007, while the Round 1 grant (Figure 3B) supported the procurement of 8.4 M treatments as of 8 May 2007 at a cost of $11.2 M. The model's ACT procurement predictions succeeded in approximating actual purchases. For the Round 4 grant, daily cumulative ACT cost predictions were significantly correlated with actual cumulative daily expenditure, with r = 0.938 (p < 0.0001). The model predicted the grant would support yearly delivery of $19.99 M worth of ACTs; in reality, $23.41 M of treatments were delivered in 2006 (error = $3.42 M), while deliveries in 2007 were on pace to reach $13.87 M. Likewise, daily cumulative ACT cost predictions were very significantly correlated with actual cumulative daily expenditure for ACT using the Round 1 grant (r = 0.945, p < 0.0001). The model predicted yearly delivery of $2.40 M in ACT; in reality, $4.03 M worth of treatments was delivered in 2004 (error = $1.6 M), $2.47 M in 2005 (error = $0.07 M), and $2.61 M in 2006 (error = $0.21 M).

## Discussion

An accurate forecast of global demand is dependent upon a thorough understanding of the availability of resources to support antimalarial procurement, in particular from the Global Fund, at present the single largest funding source. This analysis derived predictive regression models that successfully forecasted the rate and timing of malaria grant disbursement from the Global Fund to recipient countries. Additionally, it was demonstrated that smoothed disbursement rates provided accurate prediction of ACT procurement funded by specific malaria Global Fund grants in two countries. More data from other countries in different regions and epidemiological and political contexts will be necessary to further validate this approach.

**Figure 5:**
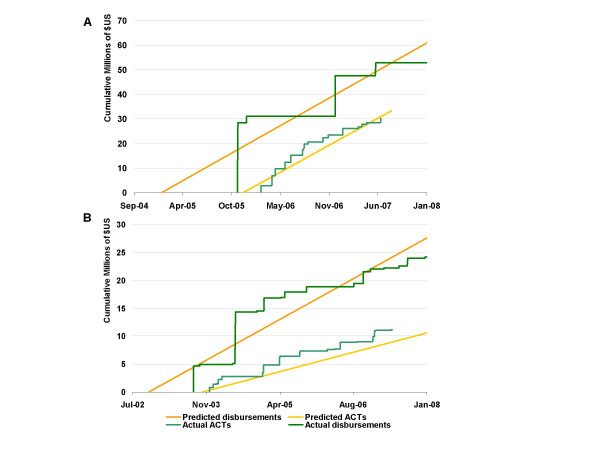
**Prediction of ACT procurement in two countries**. Validation of predicted disbursements and ACT procurement for (a) a Round 4 grant to a country in East Africa and (b) a Round 1 grant to a country in Southern Africa with actual disbursement and ACT importation records.

This investigation constructed models to predict the rate at which grants were disbursed from the Global Fund to recipient countries, rather than the exact days and magnitudes of discrete disbursements. By predicting disbursement rate, rather than discrete disbursements, it is acknowledged that precise prediction of actual quarterly disbursements – with their stochastic temporal patterning (Figure 3) – is impractical. Predicting discrete disbursements may be the ultimate goal of forecasting efforts for the purpose of intervention planning at a country level, since knowing when resources will be available is crucial to planning efforts. However, the smoothed rates forecasted here, which distribute error across time rather than attempting to estimate specific days on which disbursements occur, appear to be sufficient for general prediction of ACT procurement. In the two countries for which validation data were collected (Figure 5), this approach appears useful for forecasting the significant portion of global ACT demand driven by Global Fund grants. The rate of grant disbursement is closely tied to the rate of ACT procurement because Global Fund grants are performance-based, meaning that the disbursement slope is an indicator of grant progress. As such, if a substantial portion of a particular grant is intended for ACT procurement, a large disbursement slope may be considered indicative of successful procurement, and the magnitude and timing of that procurement can be estimated as a function of the disbursement slope.

One potential problem with using the disbursement rate as a proxy for the ACT procurement rate is that the Global Fund considers the entirety of a grant when determining performance, so an underperforming component (e.g., insecticide-treated nets) may delay disbursement of a grant even if ACT procurement is in fact on schedule. However, the subsequent funding delays also will affect all activities funded by the grant, including ACT procurement, so a slower rate of disbursement will remain associated with the procurement rate even though the causality of the association is reversed. Additionally, modeling each grant individually and aggregating for a global total allows a "portfolio" approach to forecasting, in which random error in estimation of funds in one country is balanced by the random error in another.

Some, although not all, of the factors identified here, like measures of government effectiveness and grant size, were similar to those identified in prior investigations [12,13]. Certain variables, such as national health spending, were found to be associated with disbursement in prior analyses but did not improve predictive models here, indicating that a variable may be associated with grant performance without being useful for prediction [16]. Unsurprisingly, factors found to be predictive of the rate at which funding was disbursed included several variables related to the size of the grant, including being located in East Africa (through Rounds 1–6, the average total potential size of malaria grants awarded to East African countries was US $44 million, compared to an average of $17 million for malaria grants to the rest of the world). However, even after controlling for these factors describing the grant size, additional variables related to other grant and country characteristics remained in the models as important predictive factors. Grants awarded in a later round were found on average to have larger grant disbursement rates, while countries ranked as having more corruption displayed smaller rates. Amongst other factors, the intercept of the disbursement line (timing of the initial disbursement) was predicted by the World Bank rating for government effectiveness, with more effective governments averaging an earlier start date, and the Observer compilation of human rights violations, with countries scoring worse averaging later start dates. These results appear to indicate that effective, efficient governments with respect for human rights were better able to navigate the grant signing process and to receive initial disbursements more rapidly than less effective governments.

This methodology provides a means of forecasting ACT procurement driven by Global Fund grants over their lifespan which appears to be more accurate than reliance upon planned funding, although the magnitude of the increase in predictive accuracy varies by year (Figure 4). Past experiences in ACT implementation in Sudan [20] and Kenya [21], among others, have demonstrated the unforeseen obstacles that may affect grant progress and underscore the need for approaches that incorporate such delays when forecasting resources for ACT procurement. Simple summation of planned procurement overestimated true procurement by 63% in 2005, 51% in 2006, and 17% in 2007. Recent market instability, including dramatic changes in the price of artemisinin, have been linked to overestimates of demand in past forecasts [4,22], emphasizing the need to account for the fact that actual disbursement and utilization of Global Fund grants tend to occur behind schedule [23].

Ultimately, a global forecast useful for planning drug production and artemisinin cultivation must predict demand for ACT on a product- and dosage-specific basis. The Global Fund-driven ACT funding predicted by this model provides a foundation for such a robust, accurate demand forecast. Conversion of ACT funding into product- and dosage-specific treatments, and subsequently into active pharmaceutical ingredients and artemisinin requirements, may be performed using information contained in Global Fund proposals specifying exact products to be procured, country malaria guidelines dictating public sector product choice, and epidemiological and demographic characteristics which, in the absence of other information on planned procurement, can be used to estimate the relative age-specific breakdown of dosages required. These calculations must include consideration for how the price of ACT may have decreased since the country planned its ACT procurement in its Global Fund proposal, thus altering the volume of drugs that can be purchased with the predicted disbursements.

An accurate forecast of future Global Fund supported ACT procurement is an important component of robust global demand estimation. However, estimating total global demand for antimalarials must also consider the role played by other important public sector funding organizations, including the World Bank, the United States President's Malaria Initiative, and UNITAID, as well as the growing importance of private sector procurement. Additionally, the methods described here cannot predict procurement funded by grants not yet awarded by the Global Fund. These components of the global demand require separate forecasts.

## Conclusion

This report describes an approach to predicting the size and timing of the Global Fund monetary disbursements that will continue to be used to drive the bulk of ACT purchases, at least until implementation of a global subsidy for ACT, currently being discussed by the international malaria community, becomes a reality. The evidence suggests that this approach is more accurate than forecasting based on planned funding from the grant proposals. Approaches like this one are useful tools for building comprehensive, robust models of global ACT demand that can help ensure affordable, universal access to these life-saving drugs.

## Authors' contributions

JMC participated in study conception, study design, literature review, model development, statistical analysis, analysis of results, and preparation and editing of the final report. IS participated in study conception, study design, analysis of results, and editing of the final report. MEO participated in study conception, study design, model development, analysis of results, and editing of the final report.

## Supplementary Material

Additional file 1 **Statistical models predicting the rate of grant disbursement and data of initial disbursement**. The data provided represent the final predictive regression models selected through the repeated split-sample model fitting procedure. Click here for file
