# Supplementary material for: Predicting Global Fund grant disbursements for procurement of artemisinin-based combination therapies
Source: Malar J. 2008 Oct 2;7:200. doi: 10.1186/1475-2875-7-200 (PMC2570684; doi:10.1186/1475-2875-7-200)
Supplement: Additional file 1 — Statistical models predicting the rate of grant disbursement and data of initial disbursement. The data provided represent the final predictive regression models selected through the repeated split-sample model fitting procedure. [file 1475-2875-7-200-S1.doc]

**Additional file 1**

**Statistical models predicting the rate of grant disbursement and data of initial disbursement**

Model to predict rate of grant disbursement (slope)

Slope = -**1375.98315** +**0.0006457714***P1_funding -**0.000041794***P2_agreedfunding

+**0.000153703***Total_agreedfunding +**1323.1801907***E_Africa +**1097.5916899***Round +**2142.2676977***CorruptionIndex

*Where:*

P1_funding = Total possible funding for phase one of the grant

P2_agreedfunding = Current amount of funding agreed for phase two of the grant

Total_agreedfunding = Current amount of funding agreed for phase one and two of the grant

E_Africa = Dummy variable equaling one if the country is in East Africa, zero otherwise

Round = The funding round in which the grant was awarded

CorruptionIndex = World Bank Control of Corruption Index, which ranges from -2.5 to 2.5, with higher values indicating greater control of corruption

*For countries where the Control of Corruption Index is unavailable, the following model is fit instead:*

Slope = -**2846.027808** +**0.0006588423***P1_funding -**7.832576E-6*** P2_agreedfunding

+**0.0001247472***Total_agreedfunding +**2281.8716607***E_Africa +**1076.5507446***Round;

Model to predict date of initial disbursement (intercept)

Intercept = ApprovalDate -**14.22747327**+**105.29174351***SEARO -**9.846553891***AFRO -**16.81450786***AMRO

+**21.601601418***GrantLength +**34.554328392***MalariaGrants -**35.13230493***Round

-**93.12534089***GovEffectiveness +**4.2074429409***HumanRights

*Where:*

SEARO = Dummy variable equaling one if country is in the WHO Southeast Asia region, zero otherwise

AFRO = Dummy variable equaling one if country is in the WHO African region, zero otherwise

AMRO = Dummy variable equaling one if country is in the WHO American region, zero otherwise

GrantLength = Number of years grant is expected to span (obtained from grant proposal)

MalariaGrants = Number of malaria grants the country has received from the Global Fund

GovEffectiveness = World Bank Government Effectiveness Index, which ranges from -2.5 to 2.5, with higher values indicating greater effectiveness

HumanRights = Observer Human Rights Index (1999), compiling the number of human rights abuses by country, weighted by the Human Development Index

*For countries where the Government Effectiveness or Human Rights Indices are unavailable the following is used:*

Intercept = ApprovalDate +**143.6602363** +**74.238979764***SEARO -**28.1634033***AFRO -**34.23449025***AMRO

+**17.913605752***GrantLength +**16.317714325***MalariaGrants -**42.1556712***Round

+**1.5964304E-6***Total_agreedfunding
